# Root canal curvature influences uncontrolled removal of dentin and cleaning efficacy after ultrasonic activation

**DOI:** 10.1590/0103-6440202405611

**Published:** 2024-03-22

**Authors:** Hermano Camelo Paiva, Elaine Faga Iglecias, Laila Gonzales Freire, George Táccio de Miranda Candeiro, Basílio Rodrigues Vieira, Frederico Barbosa de Sousa, Giulio Gavini

**Affiliations:** 1 Department of Restorative Dentistry, School of Dentistry, University of São Paulo, São Paulo, Brazil.; 2 Universitary Center Unichistus, Fortaleza, Ceará, Brazil; 3 Federal University of Paraíba, João Pessoa, Paraíba, Brazil

**Keywords:** Ultrasonic Activation, Endodontic Therapy, Micro-Computed Tomography

## Abstract

This study evaluated the correlation between root canal curvature and the effects of ultrasonic irrigation in the following parameters: volume of uncontrolled dentin removal (UDR_Vol_), maximum depth of dentin defects, removal of accumulated hard tissue debris (AHTD), and canal transportation in prepared curved root canals. Twenty-four human permanent mandibular molars were divided into two groups according to root canal curvature: moderate curvature (MC: mean 25°); and severe curvature (SC: mean 48°). The specimens were scanned using an X-ray microcomputed scanner (Skyscan 1172) before and after cleaning and shaping and after the final irrigation protocol with ultrasonic irrigation. There was a moderate correlation between the degree of root canal curvature and the volume of remaining AHTD (p<0.05) and between the degree of root canal curvature and maximum depth of defects due to uncontrolled removal of dentin (p<0.05). The teeth in the SC group had a greater maximum depth of defects on the dentin wall in the apical third than the teeth in the MC group (p <0.05). Both groups had a significant reduction of AHTD in all canal thirds, but the amount of remaining AHTD in the middle and apical thirds and the whole canal was significantly greater in the SC than in the MC group (p <0.05). Canal transportation was not influenced by the canal curvature in all thirds (p >0.05). This study concluded that root canal curvature affects significantly the uncontrolled removal of dentin and remaining AHTD volume after the final irrigation protocol with ultrasonic irrigation.

## Resumo

Este estudo avaliou a correlação entre a curvatura do canal radicular e os efeitos da irrigação ultrassônica nos seguintes parâmetros: volume de remoção não controlada de dentina (UDR_Vol_), profundidade máxima do desgaste de dentina, remoção de debris de tecido duro acumulado (AHTD) e transporte em canais radiculares curvos. Vinte e quatro molares inferiores humanos permanentes foram divididos em dois grupos de acordo com a curvatura do canal radicular: curvatura moderada (MC: média de 25°); e curvatura acentuada (SC: média de 48°). Os espécimes foram digitalizados usando um scanner microcomputador de raios X (Skyscan 1172) antes e depois da limpeza e modelagem e após a irrigação ultrassônica. Houve uma correlação moderada entre o grau de curvatura do canal radicular e o volume de AHTD remanescente (p<0,05) e entre o grau de curvatura do canal radicular e a profundidade máxima de defeitos devido à remoção descontrolada de dentina (p<0,05). Os dentes do grupo SC apresentaram maior profundidade máxima de defeitos na parede dentinária no terço apical do que os dentes do grupo MC (p<0,05). Ambos os grupos tiveram uma redução significativa de AHTD em todos os terços do canal, mas a quantidade de AHTD remanescente nos terços médio e apical e em todo o canal foi significativamente maior no grupo SC do que no grupo MC (p <0,05). O transporte do canal não foi influenciado pela curvatura do canal em todos os terços (p > 0,05). Este estudo concluiu que a curvatura do canal radicular afeta significativamente a remoção descontrolada de dentina e o volume remanescente de AHTD após a irrigação ultrassônica.

## Introduction

One of the most important stages of root canal preparation is irrigation and aspiration ^1^. Conventional irrigation with only a syringe and needle may be ineffective and not produce an adequate distribution and penetration of the irrigating solution, which might affect root canal cleaning and disinfecting, particularly in curved canals ^2^ ^,^ ^3^.

Studies using micro-computed tomography (micro-CT) showed that after root canal cleaning and shaping, hard tissue debris with a density similar to that of dentin is found in root canal space. This suggests that dentin chips are produced and form a compact filling inside the root canal system called accumulated hard tissue debris (AHTD) ^4^ ^,^ ^5^.

The efficacy of different irrigation methods may be improved using agitation techniques that increase the reach of the irrigant solutions and result in better removal of AHTD ^5^ and disinfection of the root canal system ^6^.

Ultrasonic activation, the most common method to increase irrigant efficiency, has been described as passive when an ultrasonic insert oscillates freely without any contact with the root canal walls ^7^ ^,^ ^8^. However, recent studies found that ultrasonic irrigation may result in uncontrolled removal of root canal dentin (URD), even when using smooth files or inserts ^8^ ^,^ ^9^ ^,^ ^10^. The type of ultrasonic insert, its activation time, and its positioning inside the canal may affect the URD and its intensity ^8^ ^,^ ^9^.

Studies about URD after ultrasonic irrigation are scarce in the literature ^8^ ^,^ ^9^ ^,^ ^10^ and do not define whether the degree of root canal curvature might affect URD, as well as the mechanical efficiency of ultrasonic irrigation and canal transportation, which should, therefore, be investigated.

This study used a micro-CT to evaluate the correlation between the degree of root canal curvature and the volume and maximum depth of URD, the percentage of remaining AHTD, and the canal transportation after ultrasonic irrigation. The null hypothesis was that the degree of root canal curvature would not affect the results of uncontrolled dentin removal, the amount of AHTD removed, or canal transportation from mesial canals of mandibular molars.

## Material and methods

### Tooth selection and sample preparation

This study was approved by the local ethics in research committee (approval no.: 01117718.2.0000.0075). The sample size was defined as 12 specimens per group using the GraphPad Prism software (GraphPad Software, San Diego, SC) and a two-tailed test for two independent groups; alpha error was set at 5%, and test power, was at 80%. All procedures were carried out by an endodontic specialist with more than 7 years of clinical experience.

One hundred and twenty-seven extracted mandibular molars were scanned using an X-ray micro-CT scanner (Skyscan 1172, Bruker microCT, Kontich, Belgium). The teeth were provided by the Biobank of human teeth from the School of Dentistry of the University of São Paulo. The teeth were stored for 30 days before being used in the study and only mesial roots were used. To ensure homogeneity between groups, twenty-four mandibular molars were selected according to the following inclusion criteria: fully formed apex, no cracks or fractures, and no resorptions, calcifications, or previous endodontic treatment. Morphological characteristics and internal anatomy were also standardized: initial canal volume, degree of root canal curvature, type II configuration according to Vertucci’s classification ^11^, and type I configuration according to Fan’s isthmus classification ^12^. Root canal curvatures were measured by the Schneider 1971 method ^13^. Then, the teeth were paired according to the degree of canal curvature and divided into two groups (n=12): moderate curvature (MC: up to 30^o^) and severe curvature (SC: over 30^o^). Tooth length was standardized at 17 mm by sectioning the occlusal surfaces using a diamond disc (Buehler, Lake Bluff, IL). Root canals in which patency was not established were excluded and replaced.

### Cleaning and shaping

Access was achieved using #1014 and #3081 diamond burs (KG Sorensen, São Paulo, Brazil). The root canals were explored using #10 hand K-files (Dentsply Maillefer, Ballaigues, Switzerland) inserted until the tip was visualized at the apical foramen under an operative microscope (DFV, São Paulo, Brazil) at an 8x magnification. Working length (WL) was set at 1 mm short of the apical foramen.

The roots were covered with polyvinyl siloxane (Speedex; Coltene, Cuyahoga Falls, OH) creating a closed-end system to avoid passive extrusion of the irrigant.

A #15 hand K-file (Dentsply Sirona, Ballaigues, Switzerland) was used to establish the glide path, and a 30/.10 file (EasyEndo, Belo Horizonte, Brazil), to prepare the cervical area. After that, the root canals were prepared to WL using Mtwo files (VDW GmbH, Munich, Germany): 10/.04, 15/.05, 20/.06, 25/.06, 30/.04 and 35/.04. The canals were irrigated with 5 mL of 2.5% NaOCl (Formula & Action, São Paulo, Brazil) after each file change using a 5 mL syringe (Ultradent, South Jordan, UT) and a 30-G NaviTip needle (Ultradent, South Jordan, UT). After preparation, the mesial canals were irrigated with 5 mL 17% EDTA (Formula & Action, São Paulo, Brazil), which remained inside the canals for 2 minutes, and 5 mL 2.5% NaOCl. The irrigant was aspirated using a capillary tip (Ultradent, South Jordan, UT) and dried with 35/.04 paper points (VDW GmbH Munich, Germany). The condensation silicone was removed, and the teeth were repositioned in the carrier port for post-preparation scanning.

### Ultrasonic irrigation

All mesial canals were irrigated with 2mL 1% NaOCl followed by activation of the irrigant for 20 seconds with a 20/.01 smooth ultrasonic insert (Irrisonic, Helse Dental, Santa Rosa de Viterbo, Brazil) coupled to an ultrasonic piezoelectric device, at 20% power setting (PiezonMaster, PM200-SEM, Satelec Acteon Group, Mérignac, France). This procedure was repeated 2 more times, once with 2 mL 17% EDTA and once with 2 mL 1% NaOCl ^10^ ^,^ ^13^.

The ultrasonic insert was pre-curved individually for each root canal and positioned buccolingually ^14^ to oscillate by using in-and-out movements, respecting the maximum distance of 2 mm from WL. After the final irrigation protocol with ultrasonic irrigation, each canal was irrigated with 5 mL of 2.5% NaOCl. Finally, the canals were aspirated with a capillary tip suction cannula and dried with 35/.04 sterile absorbent paper points. The condensation silicone was removed, and the teeth were repositioned in the carrier port for post-irrigation scanning.

### Micro-CT Scanning

The teeth were scanned before and after canal preparation and after the final irrigation protocol with ultrasonic irrigation using a Skyscan 1172 micro-CT scanner (Bruker microCT, Kontich, Belgium) at 90 kV, 278 mA, 360 rotation, 0.5° rotation stepsize, and voxel size of 17.42-µm. After image acquisition, the NRecon 1.6.10.4 software (Bruker, Kontich, Belgium) was used to reconstruct cross-sectional images. Reconstruction parameters were adjusted for noise suppression using the following fine-tuning functions: Gaussian smoothing filter (kernel = 2), beam-hardening correction of 40%, post-alignment of 0.50 to compensate for possible misalignment during acquisition, and ring artifacts correction of 10.

### Image Analysis

The DataViewer 1.5.1 software (Bruker-microCT, Kontich, Belgium) was used for the co-registration of the sets of images so that they could be aligned in the same spatial position. The CTan 1.16.4.1 (Bruker-microCT, Kontich, Belgium) and the CTvol 2.3.2.0 (Bruker-microCT, Kontich, Belgium) applications were used to calculate quantitative data and create the 3D models. The volume of interest in each specimen was defined as the area from the furcation to the apex of the mesial roots. The grayscale level necessary to identify dentin before and after cleaning and shaping and after final irrigation was determined on a density histogram using a global thresholding method. Custom processing tools and arithmetic operations were used to segment images according to the parameters under analysis.

### The volume of uncontrolled removal of dentin (URD_Vol_)

The volume of uncontrolled removal of dentin (URD_Vol_) when using an ultrasonic insert was measured using a set of morphological operations by superimposing baseline, post-preparation, and post-irrigation images. The URD_Vol_ was calculated by subtracting the volume of the canal after preparation (C), without debris, from the volume of the canal after ultrasonic irrigation (D), also without debris, using the following formula:

$${URD}_{Vol}=(D-B)-(C-A)$$

### Maximum depth of dentin defects

**Figure 1:**
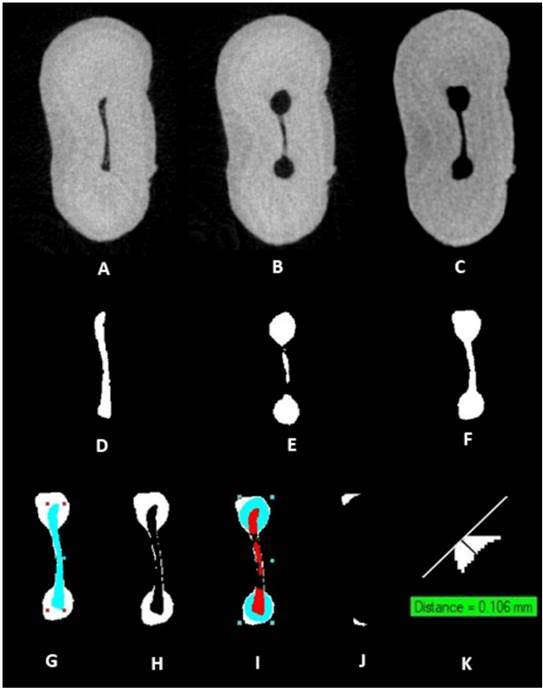
Microtomographic sections of the apical third of a sample, demonstrating Uncontrolled Dentin Removal analysis. (A) Cross-sectional image showing the preoperative root canal. (B) Cross-sectional image showing the prepared canal. (C) Cross-sectional image showing the canal after ultrasonic activation. (D) Segmented preoperative canal. (E) Segmented prepared canal. (F) Segmented root canal after ultrasonic activation. (G, H, and I) Superimposition and subtraction of D, E, and F; (J) Segmented Uncontrolled Removed Dentin. (K) Measurement of maximum depth of defects.

The maximum depth of dentin defects on the canal wall was calculated using the location of the canal wall after preparation as a reference. A tangent to the most external point of defect was drawn, followed by another line, perpendicular to this tangent. The *Measure* tool of the Ctan software was used to measure the distance between the most external point of defect to the canal wall after preparations, as shown in Figure 1.

### Accumulated Hard Tissue Debris (AHTD)

AHTD volume after preparation (A) and AHTD volume remaining after the final irrigation protocol with ultrasonic irrigation (B) were calculated using a series of morphological operations between the superimposed cross-sectional images, as described in a previous study ^5^. All material with a density similar to that of dentin in regions previously occupied by air in the preoperative canal space was identified as AHTD on the postoperative images, and the amount of AHTD was the intersection between the images acquired before and after canal preparation. The remaining AHTD volume after final irrigation was measured using the superimposition of the original post-irrigation images and the binary image of the debris after preparation. The percentage of remaining AHTD was calculated using the following formula:

% of remaining AHTD = (B x 100)/A

### Root canal transportation

Root canal transportation after the final irrigation protocol with ultrasonic irrigation was analyzed three-dimensionally using the values of the x, y, and z planes. For that, a centroid was defined as the center of gravity of each cross-sectional image of the canal and the connection of these centers along the z-axis. The canal transportation (Tp) was determined according to the comparison of the centers of gravity of the canals after preparation and after irrigation, using the following formula ^15^:

$${Tp}^{2}=\;{\left(X_{1}-X_{2}\right)}^{2}\;+\;{\left(Y_{1}\;-{\;Y}_{2}\right)}^{2}\;+\;{\left(Z_{1}-Z_{2}\right)}^{2}$$

The URD_Vol_, maximum depth of defect on the canal walls, percentage of remaining AHTD, and canal transportation were calculated separately in the apical, middle, and cervical thirds of each canal.

### Statistical analysis

The Jamovi 1.6 software was used for statistical analyses. The Man-Whitney test was used when there was no normality and homogeneity of variance and the Student *t* test when there were normal distributions and homogeneity of variance. Paired *t*-tests and the Wilcoxon test were used for intragroup comparisons when there were normal or non-normal distributions, respectively. The Person and the Spearman tests were used to analyze the correlation between the degree of canal curvature and the remaining debris volume and between the degree of canal curvature and URD_Vol_.

## Results

Samples were distributed homogeneously according to the baseline values of canal volume and the values of AHTD volume after cleaning and shaping, without any significant differences between groups (p>0.05) (Table 1). The degree of curvature in the moderate curvature group ranged from 18° to 30° (mean 25°) and in the severe curvature from 38º to 58º (mean 48º).

**Table 1:** Mean ± standard deviation values of baseline canal volume and median (minimum and maximum) values of AHTD volume after instrumentation.

	Severe curvature	Moderate curvature	p-value
Baseline canal volume (Mean ± SD)	5.95 ± 2.59	7.62 ± 3.41	0.228
AHTD volume after instrumentation Median (Min-Max)	0.262 (0.133-0.791)	0.376 (0.0786 - 0.718)	0.552

### The volume of uncontrolled removal of dentin (URD_Vol_)

The analysis of uncontrolled dentin removal after the final irrigation protocol with ultrasonic irrigation of curved canals revealed significant differences in volume between canal thirds. The volume of removed dentin was the greatest in the apical third, regardless of group (p<0.05). There were no significant differences in the volume of removed dentin between groups (Table 2).

**Table 2:** Median (minimum and maximum) values of volume, depth of defects, and root canal transportation after ultrasonic irrigation in the cervical, middle, and apical thirds and in the whole canal.

Uncontrolled removal of dentin and root canal transportation			
		Moderate Curvature	Severe Curvature
		Median (min-max)	Median (min-max)
Total	Volume (mm³)	0.0654 (0.0370-0.423)^a^	0.128 (0.0307-0.189)^a^
Cervical Third	Volume (mm³)	0.004(0.00-0.139)^a^	0.0114 (0.00-0.0512)^a^
	Maximum depth (mm)	0.049(0.00-0.0188)^a^	0.07 (0.00-0.123)^a^
	Transportation (mm)	0.0167(0.00406-0.448)^a^	0.0142 (0.00624-0.0539)^a^
Middle Third	Volume (mm³)	0.0219(0.00-0.223)^a^	0.0303(0.00215-0.104)^a^
	Maximum depth (mm)	0.129(0.00-0.321)^a^	0.141(0.0210-0.405)^a^
	Transportation (mm)	0.0226(0.0080-0.458)^a^	0.0318(0.00710-0.103)^a^
Apical Third	Volume (mm³)	0.0324(0.01-0.208)^a^	0.0732(0.0115-0.112)^a^
	Maximum depth (mm)	0.160(0.055-0.411)^a^	0.358(0.084-0.592)^b^
	Transportation (mm)	0.0513(0.0141-0.412)^a^	0.0571(0.0148-0.129)^a^

Different lowercase letters on each line indicate statistically significant differences between groups (p <0.05).

### Maximum depth of dentin defects

**Figure 2:**
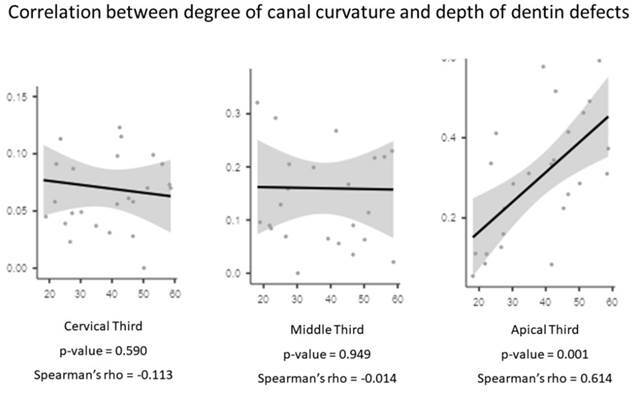
Graphics of correlation between degree of canal curvature and depth of dentin defects.

The depth of defect was significantly greater in the apical thirds of the SC group (Table 2). The Spearman correlation test revealed a moderate association between the degree of root canal curvature and the depth of defects in the apical third (Spearman’s rho = 0.614) (Figure 2).

### Accumulated Hard Tissue Debris (AHTD)

**Figure 3:**
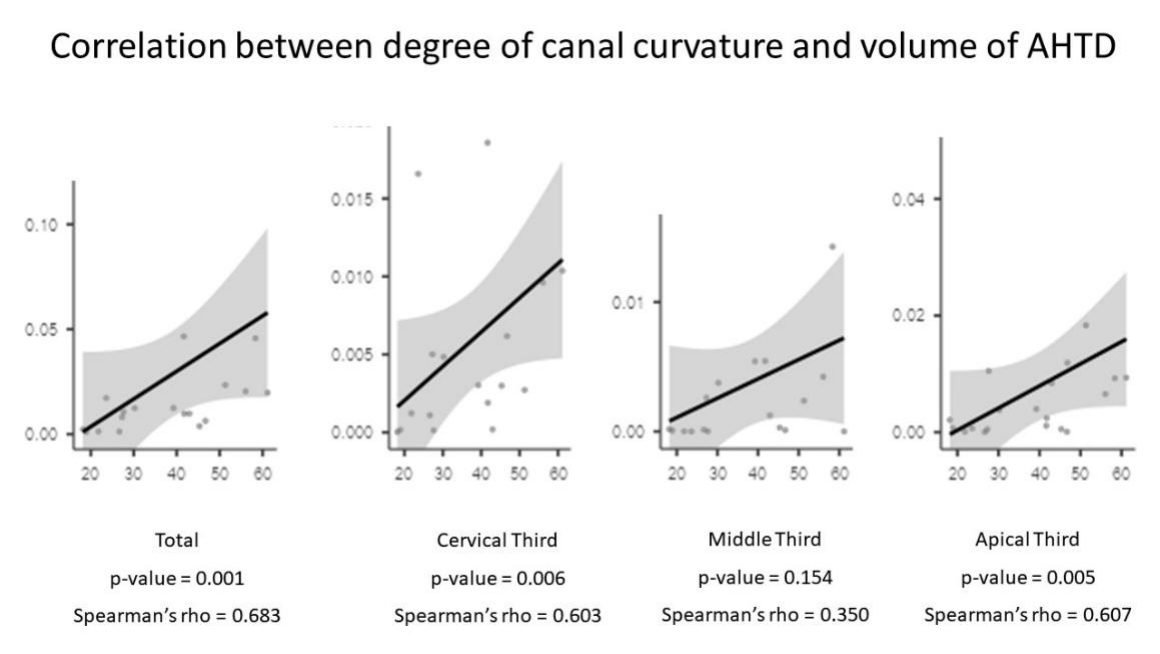
Graphics of correlation between the degree of canal curvature and volume of remaining debris.

The Spearman correlation test revealed a moderate association between the degree of root canal curvature and the volume of remaining AHTD in the whole canal (Spearman’s rho = 0.683) and the cervical (Spearman’s rho = 0.603) and apical thirds (Spearman’s rho = 0.607) (Figure 3). Intragroup comparisons of AHTD volume before and after the final irrigation protocol with ultrasonic irrigation in both groups revealed that the reduction of AHTD was significant after ultrasonic irrigation (p<0.05) (Table 3). The comparison of these parameters between groups revealed significant differences in the cervical and middle thirds, but not in the apical third (Table 3). The percentage of remaining AHTD was significantly higher in all thirds in the SC group (p<0.05) (Table 3).

### Root Canal transportation

Root canal transportation was not significantly different in any canal third (p>0.05) (Table 2), and there was no correlation between the degree of curvature and canal transportation.

**Table 3:** Median (minimum and maximum) values of AHTD and percentage of remaining AHTD after ultrasonic irrigation in the cervical, middle, and apical thirds and in the whole canal.

AHTD (mm³)			
		Moderate Curvature	Severe Curvature
		Median (min-max)	Median (min-max)
Total	Post Instrumentation	0.376 (0.133-0.791)^Aa^	0.262 (0.0786-0.718)^Aa^
	Post Ultrasonic Irrigation	0.0081 (0.00-0.0172)^Ba^	0.0204 (0.0038-0.165)^Bb^
	% of remaining AHTD	1.98 (0.16-8.03)^a^	7.2 (3.85-40.3)^b^
Cervical Third	Post Instrumentation	0.142 (0.0387-0.257)^Aa^	0.166 (0.0312-0.321)^Aa^
	Post Ultrasonic Irrigation	0.00123 (0.00-0.0166)^Ba^	0.00961 (0.00-0.102)^Bb^
	% of remaining AHTD	1.06 (0.01-12.5)^a^	7.38 (0.247-60.7)^a^
Middle Third	Post Instrumentation	0.123 (0.0033-0.444)^Aa^	0.0725 (0.0108-0.243)^Aa^
	Post Ultrasonic Irrigation	0.00013 (0.00-0.00542)^Ba^	0.00423 (0.00-0.0509)^Bb^
	% of remaining AHTD	0.04 (0.09-6.3)^a^	7.11 (0.09-52.4)^b^
Apical Third	Post Instrumentation	0.0347 (0.0063-0.205)^Aa^	0.0231 (0.0097-0.153)^Aa^
	Post Ultrasonic Irrigation	0.00072 (0.00-0.0105)^Ba^	0.00838 (0.00-0.0591)^Ba^
	% of remaining AHTD	2.6 (0.05-16.8)^a^	22.0 (0.7-40.5)^b^

Different uppercase letters on each column indicate statistically significant differences in the same group after ultrasonic irrigation (P <0.05).

Different lowercase letters on each line indicate statistically significant differences between groups (p <0.05).

## Discussion

**Figure 4:**
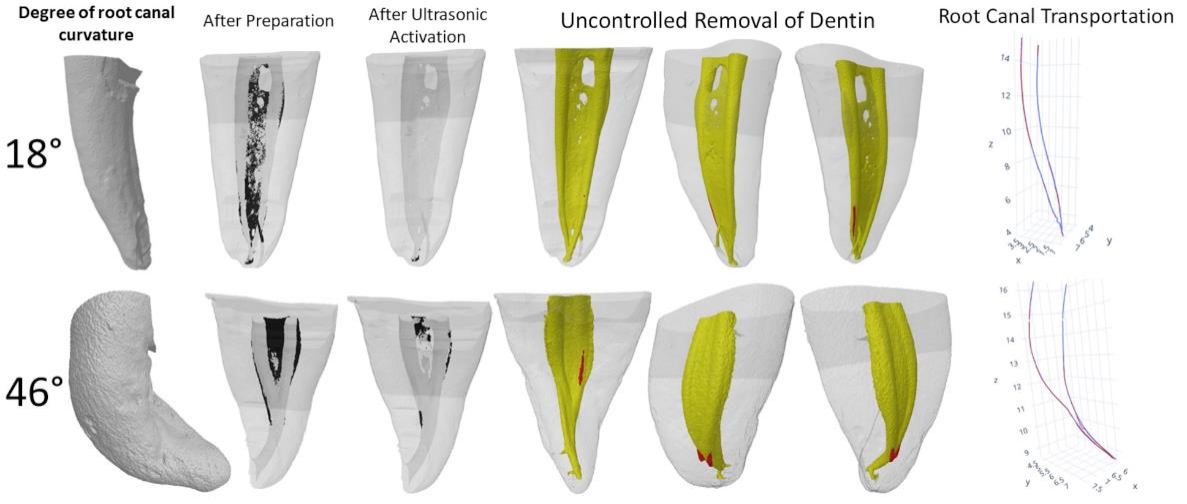
Three-dimensional reconstructions of micro-CT scans of the mesial root canal systems after preparation and after ultrasonic activation. AHTD after preparation and remaining AHTD after ultrasonic activation are represented by black areas. The uncontrolled removal of dentin is represented by red areas. A graph illustrating root canal transportation evaluated by the superimposition of the gravity center before (blue line) and after ultrasonic activation (red line).

Ultrasonic irrigation is the most used method to improve root canal system cleaning, given the known shortcomings of conventional irrigation, especially in curved canals ^3^ ^,^ ^16^. Therefore, the present study proposed to evaluate whether the degree of canal curvature influences the result of ultrasonic irrigation in the face of the following parameters: uncontrolled removal of dentin, accumulated hard tissue debris removal, and canal transportation. The null hypothesis was rejected because the results indicated that greater root canal curvatures negatively affect the maximum depth of dentin defect in the apical third, as well as the efficiency of AHTD removal after the final irrigation protocol with ultrasonic irrigation. In the severe curvature group, more AHTD remained in all thirds of the root canal when compared to the moderated curvature group (Figure 4). One of the possible explanations is that root canal curvature may block the flow of irrigating solution to the apical third ^3^ ^,^ ^17^.

This study evaluated *ex vivo* the side effects of activated ultrasonic irrigation comparing root canals with moderate and severe curvatures. To evaluate canal curvature, we employed the method suggested by Schneider, 1971, which categorized curvatures exceeding 25 degrees as severe. However, in our current investigation, we have delineated two distinct groups: one encompassing canals displaying moderate curvature, up to 30o, and the other comprising canals manifesting severe curvature, above 30o. This was based on the evolution of Nickel-Titanium (NiTi) alloys and instruments in recent years, which facilitated the preparation of canals that were previously considered more difficult to prepare with stainless steel hand instruments. Other studies using straight ^9^ or curved canals ^8^ ^,^ ^10^ have already demonstrated that ultrasonic irrigation may lead to uncontrolled dentin removal in root canals.

The greatest defect of ultrasonic irrigation was found at the apical third of the root canal, and the volume of uncontrolled dentin removal was significantly different between root canal thirds. Dentin defect was the greatest in the area closest to the apex, regardless of the degree of curvature. This difference may be associated with the fact that the tip of the insert is placed in the apical region and may be positioned against the canal walls in root canals with a greater degree of curvature, tending to return to its original shape due to its reduced flexibility.

Still, in the apical third, there was a moderate correlation between the degree of canal curvature and the maximum depth of the defect, which indicates that the greater the degree of curvature, the deeper the defect in this third of the root canal. Moreover, depths of defects were significantly greater in the apical third of the canals in the SC group than in the MC group (p<0.05). Tapered ultrasonic inserts have a greater displacement amplitude near the free tip, which decreases as the taper increases. This fact may also have contributed to greater dentin wear in the apical region ^18^ ^,^ ^19^.

The stainless steel ultrasonic insert used (Irrisonic, Helse Dental, Santa Rosa de Viterbo, Brazil) is smooth, with a small diameter and taper (# 20/.01), and may be pre-curved. However, the reduced flexibility of its metal alloy possibly contributed to the occurrence of dentin defects. In addition, the frequency of secondary oscillation, which results from the reflex of insert vibration due to the physical contact with the hard tissue, is dependent on file stiffness ^19^: the stiffer the ultrasonic insert, the faster it may return to the canal wall during oscillation ^19^ ^,^ ^20^. Thin and flexible inserts can also be used to mechanically activate the irrigants in root canals with an acute angle of curvature because they can reach further into the apical, such as Easy Clean (EC) (Easy Equipamentos Odontológicos, Belo Horizonte, Brazil) ^10^, XP-Endo Finisher (XPF) (FKG, La Chaus-de Fonds, Switzerland) ^21^, or Eddy (VDW, Munich, Germany), which may be also safer for similar cases ^10^. Despite this, the effectiveness of these devices regarding root canal cleaning is still controversial when compared to ultrasonic agitation, and most of them did not assess uncontrolled wear on the canal wall ^22^.

Rodrigues et al. (2021) ^10^ found that sonic activation of irrigants in curved canals with a polymeric tip, Eddy (VDW, Munich, Germany) caused minimum removal of dentin, ranging from 10 to 50 μm, while ultrasonic irrigation reached values of uncontrolled dentin removal up to 440 μm. In the present study, the largest defect in the apical third measured 591μm (0,591 mm), which may not be large enough to reduce the resistance of root canal dentin, or promote significant canal transportation, but may affect the quality of the obturation.

In the present study canal transportation was performed in three dimensions by comparing each voxel slice of the entire volume before and after the final irrigation protocol with ultrasonic irrigation, instead of the classically 2-dimensional way in predetermined sections ^10^ ^,^ ^23^. Therefore, it was possible to obtain the results of the centers of gravity, connected along the z-axis, indicating the 3D trajectory of the root canal and possible changes after ultrasonic irrigation. No significant differences were found in root canal transportation between the groups, nor was there a correlation between the degree of curvature and root canal transportation. This result corroborates other studies that showed that the use of ultrasonic inserts does not cause significant root canal transportation, as long as they are used prebend ^24^ ^,^ ^25^.

Although root canal transportation is not significant, the ledge formed by the action of an ultrasonic insert may block the apical insertion of the irrigation needle and complicate the adaptation of the gutta-percha cone during obturation ^26^. Wu et al. ^27^ found that apical transportation greater than 0.3 mm may affect apical sealing negatively. Further studies should evaluate the impact of these defects on the quality of sealing and obturation.

This study used the irrigation protocol suggested by van de Sluis et al. 2010 ^13^, in which three refreshment/activation cycles have a cumulative effect ^13^. The same irrigation protocol was also used by Rodrigues et al. 2021, although the authors were not concerned about directing the ultrasonic insert towards the isthmus. In the present study, the ultrasonic insert was used in the same direction as the isthmus. The oscillation of the ultrasonic insert towards the groove is more efficient in removing AHTD than the oscillation perpendicular to the groove. This may be explained by the high-velocity jet of irrigant in a single direction at the tip after oscillation, in contrast with a slow insert oscillation and inflow in the perpendicular direction ^14^.

There was a moderate correlation between the degree of curvature and the volume of remaining AHTD in the cervical and apical thirds and the whole canal (p<0.05). In addition, the SC group had a significantly greater volume and percentage of remaining AHTD than the MC group. Although the contact of the ultrasonic file with the canal walls does not block file oscillation completely ^8^ ^,^ ^20^, our results may be associated with the degree of root curvature, which results in a longer contact time of the insert with the canal walls and a lower micro-vibration capacity. Consequently, there may be a reduction in the occurrence of stable and transient cavitation, which is fundamental for the efficacy of ultrasonic irrigation ^7^ ^,^ ^16^.

AHTD in areas that are difficult to reach may affect the quality of obturations ^28^, reduce irrigant flow ^29^, reduce the efficacy of intracanal medicaments ^30^, and leave viable microorganisms inside the root canal system ^31^. Despite these findings, no clinical study has confirmed that the use of an agitation device has an impact on the success of endodontic treatments ^32^.

The main limitation of the study is that it is an in vitro experiment. Although the results of laboratory studies should not be directly extrapolated to clinical settings, they can provide important information about some of the parameters that can interfere with the efficiency of ultrasonic irrigation. The impact of uncontrolled wear on the quality of root canal obturation must be addressed in future studies.

According to our results and other studies, ultrasonic irrigation should not be classified as passive ^8^ ^,^ ^9^ ^,^ ^20^. Therefore, current ultrasonic irrigation protocols should be revised, particularly for root canals with severe curvature.

## Conclusion

This *ex vivo* study found that root curvature affected negatively the volume and depth of defects on the root canal wall and the volume of remaining accumulated hard tissue debris after ultrasonic irrigation, with a positive correlation between these variables.
